# Alexandre da Costa Linhares (★1952 †2025)

**DOI:** 10.1590/0037-8682-0311-2025

**Published:** 2026-01-16

**Authors:** Francisco de Paula Pinheiro, Pedro Fernando Vasconcelos

**Affiliations:** 1 Instituto Evandro Chagas. Ananindeua, PA, Brasil.

A Brazilian medical researcher, Alexandre da Costa Linhares, was born in Belém, state of Pará, on March 3, 1952, and died in São Paulo, State of São Paulo, on April 13, 2025, at age 73, due to post-prostate surgery complications.

**Figure:**
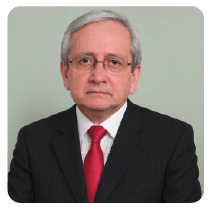

Alexandre da Costa Linhares graduated from the Faculty of Medicine of the Federal University of Pará in Belém in 1975 and completed his PhD in Parasite Biology at the Oswaldo Cruz Institute, Oswaldo Cruz Foundation (IOC/Fiocruz), Rio de Janeiro, in 2002. Linhares conducted pioneering studies on rotavirus in Brazil while still a medical student and later as an intern at the Evandro Chagas Institute (IEC) in Belém during the 1970s, where he remained until his retirement in 2019. He served as the head of the Virology section from 1987 to 2019 and as director of the IEC between 1981 and 1987. In 1975, he carried out his initial investigation of rotavirus in children with acute diarrhea treated at the Hospital da Santa Casa de Misericórdia do Pará in Belém. Rotavirus was detected in the feces of four children out of the 25 examined. The agent was identified using electron microscopy at the Bernard Nocht Institute in Hamburg, Germany, and via enzyme-linked immunosorbent assay at the IEC. In 1977, Dr. Linhares demonstrated that rotavirus was responsible for an explosive epidemic of acute diarrhea among indigenous individuals of the Tiriyó tribe, located in the northern part of the state of Pará^1^. The epidemic occurred in July and August of that year, affecting 157 (70%) of the 224 inhabitants of the main village, with one recorded death in a 1-year-old child. 

This was the first epidemic of acute diarrhea caused by rotavirus in indigenous tribes in the Americas. The epidemic affected the main village of the Tiriyó tribe, as well as other smaller villages of this indigenous group^2^. In 1978, Dr. Linhares completed an internship at the East Birmingham Hospital in the United Kingdom under the guidance of Professor Thomas Henry Flewett, during which rotavirus samples detected in the state of Pará were analyzed. One of these samples was identified as serotype 1 (Birmingham), the strain responsible for the epidemic in the Tiriyó tribe. In 1984, he completed another study in the UK in Colindale under the guidance of Professor Flewett^3^. These studies were later expanded to include other urban areas and additional viral agents associated with diarrhea, such as the Norwalk agent (currently norovirus), astrovirus, calicivirus, and others^4^. Linhares also made important contributions to clinical studies of rotavirus vaccines, and the trials conducted by the group he led were the only clinical vaccine studies conducted in Brazil^5^. Dr. Francisco Pinheiro, former director of the IEC (1979-1981), was the mentor of Dr. Linhares since his days as a medical student, and his testimony is remarkable: "Linhares had an impressive simplicity and scientific intuition, and a stoic dedication to studies with children with acute diarrhea." Professor James LeDuc of the University of Texas Medical Branch, who worked with Linhares at the IEC in Belém, stated, “He was a kind and generous person, as well as being a superb clinician and scientist.” According to virologist Pedro Vasconcelos, former director of the IEC (2014‒2019) and past president of the Brazilian Society of Tropical Medicine, “Linhares had a vocation for medical-scientific research, being a gentleman and always very attentive to the students and technicians who collaborated with him.” Alexandre Linhares also conducted important studies on acute hemorrhagic conjunctivitis, erythema infectiosum, and HTLV-I in the Amazon^6^. He also served as an editor and contributor to several scientific publications and was a member of multiple international editorial boards. His scientific legacy includes 225 articles published in journals, three books, and 39 book chapters, in addition to contributing to the training of dozens of researchers. José Paulo Gagliardi Leite, Public Health Researcher and former director of IOC/Fiocruz, and advisor of Alexandre Linhares' doctoral thesis, described their many fruitful years of collaboration as follows: “Linhares was a great friend, a unique person, owing to his professional and personal qualities. 

As a person, he was a gentleman, always available to listen and, when necessary, to speak. As a professional, he was always at the forefront of studies on gastroenteric viruses, particularly rotavirus A, one of his great passions. Not only did he describe rotavirus A for the first time in Brazil, but he was also a pioneer in clinical studies with rotavirus vaccines, including Rotarix®^7^, which has been part of the national immunization program since March 2006”. He is survived by his wife, Suely; his children Leonardo, Adriana, and Alexandre; nine grandchildren; and five siblings.
